# RACE DIFFERENCES IN WALK SPEED, CARDIORESPIRATORY FITNESS, AND MUSCLE MITOCHONDRIAL RESPIRATION

**DOI:** 10.1093/geroni/igae098.4252

**Published:** 2024-12-31

**Authors:** Paul Coen, Giovanna Distefano, Sofhia Ramos, Peggy Cawthon, Steven R Cummings, Anne B Newman, James DeLany

**Affiliations:** AdventHealth, Orlando, Florida, United States; AdventHealth, Orlando, Florida, United States; AdventHealth, AdventHealth, Florida, United States; California Pacific Medical Center, San Francisco, California, United States; California Pacific Medical Center, San Francisco, California, United States; School of Public Health, University of Pittsburgh, Pittsburgh, Pennsylvania, United States; AdventHealth, Orlando, Florida, United States

## Abstract

Older adults who self-identify as Black have a disproportionately higher incidence of mobility disability. Whether older adults who are Black also have lower fitness and mitochondrial energetics compared to those who are White has not been adequately investigated. The Study of Muscle, Mobility and Aging (SOMMA) examined 879 older adults (≥70 years old), including 116 who self-identified as Black, who could complete a 400m walk within 15 minutes. Mitochondrial respiration (MaxOXPHOS) was measured in permeabilized fibers from biopsies of the vastus lateralis. A cardiopulmonary exercise test assessed maximum oxygen consumption (VO2peak). Education, income, financial resources, race, sex and age were determined by self-report. Propensity score matching was used to match Blacks with Whites with a 1:1 ratio. Black (n=90) and White (n=90) groups were well matched for age, sex, SOMMA multimorbidity index, BMI, muscle mass, physical activity, marital status, educational achievement, and whether financial needs were met (all p>0.05). Despite being well matched, those who identified as Black had a slower 400m walking speed (0.97 vs. 1.03 m/s, p=0.014), lower MaxOXPHOS (50.8 vs. 60.9 (pmol/(s*mg)), p< 0.001), and lower cardiorespiratory fitness (1391 vs 1566 ml/min, p=0.007), when compared to those who identified as White. Multivariate regression showed that VO2peak and MaxOXPHOS, but not socioeconomic factors, attenuated the race difference in 400m walking speed. In conclusion, while the etiology of race differences in mobility is multifactorial, our data indicate that muscle mitochondrial respiration and cardiorespiratory fitness may contribute to slower walking speed of individuals who identify as Black compared to White.

